# Impact of Baseline Anemia in Patients Undergoing Transcatheter Aortic Valve Replacement: A Prognostic Systematic Review and Meta-Analysis

**DOI:** 10.3390/jcm12186025

**Published:** 2023-09-18

**Authors:** Elena Jiménez-Xarrié, Lluis Asmarats, Marta Roqué-Figuls, Xavier Millán, Chi Hion Pedro Li, Estefanía Fernández-Peregrina, Juan Sánchez-Ceña, Albert Massó van Roessel, M. Luz Maestre Hittinger, Pilar Paniagua, Dabit Arzamendi

**Affiliations:** 1 Cardiology Department, Hospital de la Santa Creu i Sant Pau, Biomedical Research Institute (IIB Sant Pau), 08025 Barcelona, Spain; 2 Biomedical Research Institute Sant Pau (IIB Sant Pau), 08025 Barcelona, Spain; 3 Anesthesiology Department, Hospital de la Santa Creu i Sant Pau, Biomedical Research Institute (IIB Sant Pau), 08025 Barcelona, Spain; 4 Centro de Investigación Biomédica en Red de Enfermedades Cardiovasculares (CIBERCV), 28029 Madrid, Spain

**Keywords:** anemia, aortic stenosis, transcatheter aortic valve replacement, TAVR

## Abstract

Transcatheter aortic valve replacement (TAVR) is currently the treatment of choice for patients aged ≥75 years with severe aortic stenosis. Preoperative anemia is present in a large proportion of patients and may increase the risk of post-procedural complications. The purpose of this prognostic systematic review was to analyze the impact of baseline anemia on short- and mid-term outcomes following TAVR. A computerized search was performed on PubMed and Web of Science databases for studies published between January 2013 and December 2022. Primary outcomes were 30-day need for transfusion, acute renal failure, 30-day and mid-term mortality, and readmission during the first year post-TAVR. Data were analyzed via random effects model using inverse variance method with 95% confidence intervals. Eleven observational studies met our eligibility criteria and included a total of 12,588 patients. The prevalence of baseline anemia ranged between 39% and 72%, with no relevant sex differences. Patients with preprocedural anemia received more blood transfusions [OR: 2.95 (2.13–4.09)]), and exhibited increased rates of acute kidney injury [OR:1.74 (1.45–2.10)], short-term mortality [OR: 1.47 (1.07–2.01], and mid-term [OR: 1.89 (1.58–2.25)] mortality following TAVR compared with those without anemia. Baseline anemia determined an increased risk for blood transfusion, acute kidney injury, and short/mid-term mortality among TAVR recipients.

## 1. Introduction

Transcatheter aortic valve replacement (TAVR) has become the mainstay treatment in most patients with severe symptomatic aortic stenosis older than 75 years or at high surgical risk [1]. Despite a progressive decrease in the age and surgical risk of TAVR candidates over time, TAVR is often offered to elderly and frail patients with multiple comorbidities. It is estimated that 10% of adults over 65 years and up to 20% over 85 years may have some degree of anemia, defined as a hemoglobin value lower than 13 g/dL for men and 12 g/dL for women [2]. The prevalence of anemia reaches 50% among TAVR candidates (vs. 16–36% in surgical patients), with severe anemia being present in nearly one-fifth of those patients [3,4]. Anemia is a well-known predictor of mortality in patients with heart failure and carries an increased risk of morbidity and mortality following TAVR [5]. The etiologies of anemia may vary from iron deficiency to chronic kidney disease, inflammatory diseases, vitamin B12 or folate deficiency, other nutritional deficiencies, or myelodysplastic syndromes, with nearly 90% of such causes being potentially reversible before the procedure [6]. Although preprocedural anemia optimization (iron supplementation or transfusion pre-TAVR) may help reduce postprocedural transfusions and bleeding complications, the potential benefit is yet to be demonstrated. The aim of this prognostic systematic review was to offer the most up-to-date evidence on the impact and post-procedural complication rates among anemic patients undergoing TAVR.

## 2. Materials and Methods

### 2.1. Search Strategy

A computerized search of literature was performed on PubMed and Web of Science databases for studies published between January 2013 and December 2022. The following key terms were used: (“aortic stenosis” or “aortic valve stenosis”) and (“TAVI” or “TAVR” or “transcatheter valve replacement”) and (“anemia” or “iron-deficiency anemia”) and (“blood transfusion” or “mortality” or “death” or “rehospitalization” or “postoperative complications”). Observational studies in which clinical outcomes of patients undergoing TAVR were compared according to the presence of baseline anemia were included. Results were reported in accordance to the Preferred Reporting Items for Systematic Reviews and Meta-Analyses (PRISMA) guidelines [7]. The review was not registered. The PRISMA checklist has been included in Table S1.

### 2.2. Inclusion and Exclusion Criteria

Studies were considered eligible if they fulfilled all of the following criteria: (1) they reported outcomes of patients with and without baseline anemia undergoing TAVR; and (2) reported at least one of our primary endpoints: need for transfusion, acute kidney injury, 30-day and mid-term (≥12 months) mortality, and hospital readmission within the first year of the procedure. Exclusion criteria included unclear definition of anemia, pre-specified subgroups of patients, case reports, and duplicate reports.

### 2.3. Risk of Bias Assessment

The Quality in Prognostic Studies (QUIPS) tool was used to assess the risk of bias and methodological quality in the included studies [8]. This tool includes the following six domains: study participation, study attrition, prognostic factor measurement, outcome measurement, adjustment for other prognostic factors, and statistical analysis and reporting.

### 2.4. Endpoints

Definition of anemia adhered to the World Health Organization definition (<13 g/dL of hemoglobin for men and <12 g/dL for women, respectively), unless an alternative cut-off point was indicated. Primary outcomes of the systematic review were 30-day need for transfusion, 30-day acute renal failure, 30-day and mid-term mortality, and readmission during the first year of the procedure. Acute kidney injury was defined according to the Valve Academic Research Consortium-2 (change in serum creatinine or urine output within seven days post-procedure) in all studies but two, [9,10] which followed the former Valve Academic Research Consortium definition (change in serum creatinine up to 72 h) (Table S2) [11,12].

### 2.5. Statistical Analysis

Odds Ratios and 95% confidence intervals were calculated to assess the association between anemia and the endpoints of interest. When adjusted odds ratios were available from the original papers, these were used as measures of associations. When unavailable, unadjusted odds ratios were estimated from the paper tables data. Published hazard ratios were also converted to odds ratios to be pooled into analyses.

A meta-analysis was conducted under the random effects model, in order to calculate a combined OR based on the individual OR for each study, applying the inverse variance method [13].

Statistical heterogeneity was assessed through the I^2^ [14]. I^2^ values ≥ 50% were considered to be indicative of heterogeneity. Meta-analysis was conducted with the random effects model, as clinical and statistical heterogeneity was expected, given the differences in the studies’ definitions of outcomes, population and duration of follow-up, as well as their observational design.

## 3. Results

### 3.1. Search Results

**Figure 1:**
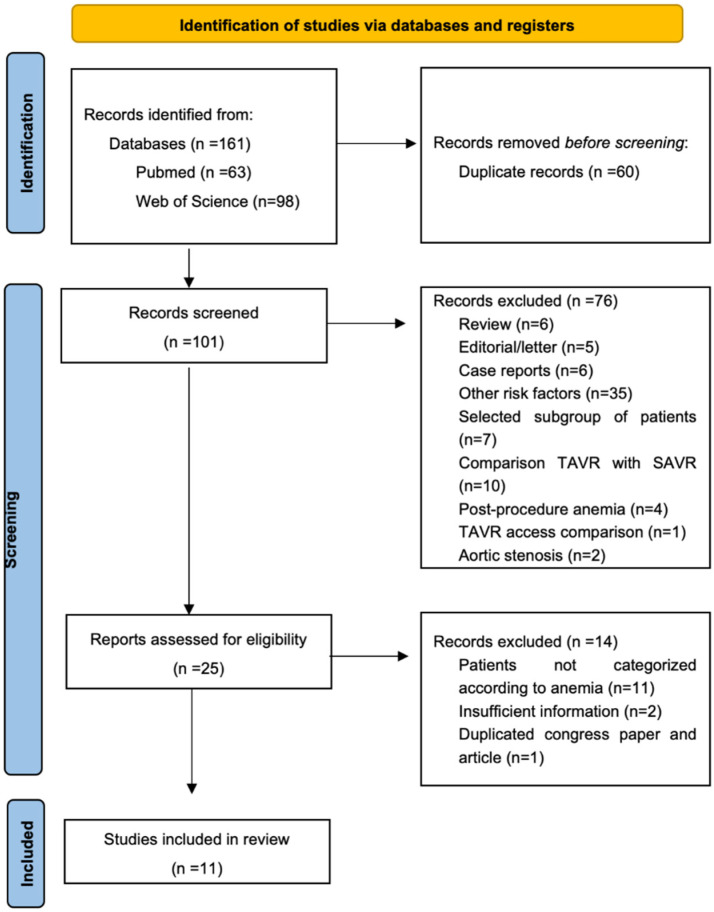
PRISMA flow diagram of studies included in this systematic review.

Our search yielded 161 results (PubMed:63 and Web of Science: 98). After removing duplicates, we screened 101 records by title and abstract, 76 were excluded according to prespecified criteria, and 25 records were screened by full-text for eligibility. Finally, 11 observational studies satisfied the selection criteria and were included in the meta-analysis (*n* = 12,588 patients). The PRISMA flow diagram of study selection is summarized in Figure 1.

### 3.2. Risk of Bias Assessment

The review included five multicenter and six single-center observational studies. A prospective design was reported in five studies [6,9,15,16,17]. All studies had a sample size of >100 anemic patients (range: 169–1335 patients). Most of the included studies were deemed to be at low or moderate risk of bias for all QUIPS domains. The detailed results of the risk of bias assessment for each of the domains according to the QUIPS tool are outlined in Table S3.

### 3.3. Clinical Characteristics

Clinical characteristics of the included studies are depicted in Table 1. The prevalence of baseline anemia according to WHO definition ranged between 39% and 72%, being higher than 50% in 9 out of the 11 studies. The most common cause of anemia in TAVR patients (~75%) was iron deficiency, although the etiology was only analyzed in two of the studies [6,18]. The average age was 82 years, and patients with preexisting anemia were significantly older than those without anemia in approximately one-third of the studies (4/11). Regarding sex distribution, no clear sex-related differences were observed.

### 3.4. Need for Transfusion

**Figure 2:**
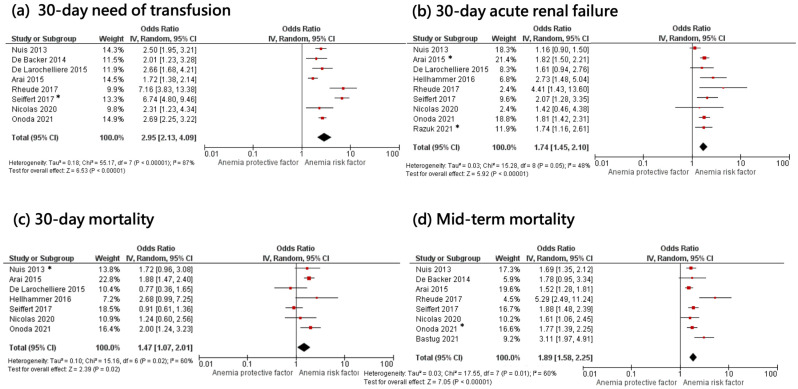
Forest plots showing pre-procedural anemia and associated odds of 30-day transfusion (**a**), acute renal failure (**b**), 30-day mortality (**c**) and mid-term mortality (**d**) [6,9,10,15,16,17,18,19,20,21,22]. Asterisk indicates reported adjusted odds ratio [10,16,21].

Need for post-procedural transfusion was available in eight studies. The transfusion rates post-TAVR in anemic patients ranged between 25% and 54%. Patients with baseline anemia more commonly required red blood cells transfusions after TAVR compared with patients without pre-operative anemia (OR: 2.95; 95% CI: 2.13–4.09; *p* < 0.001; I^2^ = 87%) (Figure 2a). In a sensitivity analysis excluding the studies with a greater effect size [18,21], anemia remained associated with significantly higher need for transfusion (OR: 2.29; 95% CI: 1.90–2.75; *p* < 0.001; I^2^ = 53%) (Figure S1).

### 3.5. Acute Kidney Injury

Acute kidney failure post-TAVR was analyzed in nine studies and was more common among patients with preexisting anemia (OR: 1.74; 95% CI: 1.45–2.10; *p* < 0.001; I^2^ = 48%) (Figure 2b).

### 3.6. Mortality

Short-term and mid-term mortality were analyzed in seven and eight studies, respectively. Mid-term follow-up was available at 1-year (*n* = 5) or ≥2 years (*n* = 4) [6,9,10,15,16,18,19,20,21]. Compared with patients who did not have pre-operative anemia, baseline anemia was associated with increased risks of 30-day (OR: 1.47; 95% CI: 1.07–2.01; *p* = 0.02; I^2^ = 60%) and mid-term mortality (OR: 1.89; 95% CI: 1.58–2.25; *p* < 0.001; I^2^ = 60%) (Figure 2c,d).

The specific causes of death were only provided by Nuis et al., 2013 [10] of which 49% were cardiovascular (similar in patients with vs. without anemia). Among non-cardiovascular causes, infection was the most common (~20%), and patients with previous anemia more frequently died because of pneumonia (11% vs. 5%, *p* = 0.024).

### 3.7. Rehospitalization

Rehospitalization was not assessed in the meta-analysis, since it was only reported in two studies. Both De Backer et al. [19] and Rheude et al. [18] identified a greater need for all-cause and heart-failure-related 1-year readmission post-TAVR, respectively, in patients with baseline anemia. However, hospital readmissions during the first year of the procedure were solely reported in these two studies and, therefore, no firm conclusions can be drawn.

## 4. Discussion

The main findings of our systematic review on the impact of preoperative anemia on post-procedural complications following TAVR can be summarized as follows: (i) preprocedural anemia is present in more than half of TAVR candidates and is often related to iron deficiency; (ii) baseline anemia carries an increased risk of post-procedural transfusion, acute kidney injury, and mortality following TAVR.

The prevalence of baseline anemia prior to TAVR in the present review varied between 39% and 72%. De Larochellière et al. [6] identified a potentially correctable cause of anemia in up to 90% of patients: 71% confirmed or possible iron deficiency (serum ferritin < 30 μg/L and/or transferrin saturation < 20%), 18% chronic renal failure, 1% vitamin B12 or folic acid deficiency, and 10% remained unexplained.

### 4.1. Clinical Impact

In the present review, preexisting anemia conferred an increased risk of poor outcomes after TAVR. Although TAVR has been associated with a lower risk of bleeding complications than surgical aortic valve replacement, the presence of lower baseline hemoglobin levels triggered an increased (~three-fold) risk of blood transfusions after the procedure compared with patients without anemia [21]. Importantly, need for transfusion has been established as an independent risk factor of poor prognosis in patients undergoing TAVR [23]. It is noteworthy that standardized transfusion thresholds for TAVR are lacking, and transfusion is generally performed on a patient-by-patient basis in contemporary practice, outlining an unmet clinical need in the management of such patients. Randomized studies are needed to establish a cut-off for red blood cells transfusion before and after TAVR.

Patients with baseline anemia exhibited a nearly two-fold (OR: 1.74) greater risk of acute kidney injury post-TAVR compared to non-anemic patients. These findings are consistent with a previous systematic review by Fowler et al. which showed a >3-fold increased risk of acute kidney injury in patients with preoperative anemia undergoing major surgery, albeit most of the studies included (14/24) were non-cardiac surgery [24]. The contributory effects of anemia on renal function are likely multifactorial, and include renal hypoxia, increased oxidative stress, and subclinical kidney disease in a significant proportion of anemic patients predisposed to renal injury. Whether this association is directly linked to lower hemoglobin levels per se or to greater requirements of red blood cells transfusion, which in turn have been linked to a pro-inflammatory state, renal artery vasoconstriction (impaired renal oxygenation and proximal tubular dysfunction), and oxidative stress, potentially enhancing the risk of acute kidney injury, remains to be determined. In addition, preexisting chronic kidney disease may predispose to episodes of acute kidney injury, although no direct association between baseline glomerular filtration rate and post-TAVR renal failure could be established [9]. Of note, two studies followed the former standardized Valve Academic Research Consortium definition, which did not consider changes in urine output or changes in serum creatinine from 72 h to 7 days [9,10]. Although these studies may have underestimated the real incidence of acute renal failure post-TAVR, the same definition was applied in both groups (patients with and without anemia) and therefore may not have influenced the results of the present study. Future prospective studies are warranted to confirm the potential effect of hemoglobin on changes in creatinine levels among patients undergoing TAVR.

Baseline anemia and lower baseline hemoglobin levels were associated with an increased risk of short and mid-term mortality after TAVR. This is of major importance when assessing the risk-benefit of any intervention, since procedural futility is often defined as death occurring less than one year after TAVR. Importantly, anemia may be considered a surrogate marker for underlying overt or subclinical conditions such as frailty, sarcopenia, or poor nutritional status, which have been associated with increased mortality post-TAVR [25]. Likewise, anemia is a common comorbidity of kidney disease, gastrointestinal, or inflammatory pathologies often encountered in elderly patients, which may negatively impact clinical outcomes by themselves [26]. Overall, the mechanisms underlying the potential association between anemia and mortality remain unknown, and future studies are needed to increase our knowledge on this matter.

Data on the impact of anemia on readmission for heart failure are scarce, since most studies have assessed the Valve Academic Research Consortium-2 efficacy endpoint (combination of death and hospital readmission for worsening heart failure) but not rehospitalization as an independent outcome itself [11]. In this review, these data were solely reported in two studies which suggested an increased risk of readmission in anemic patients [18,19]. Of note, anemia can result in an increased output state precipitating heart failure decompensation. Several studies have reported anemia as an independent predictor of early (30-day) heart failure readmission following TAVR [27,28]. Furthermore, overload due to blood transfusion during TAVR hospitalization could predispose to heart failure exacerbation and readmission during the first year of the procedure.

### 4.2. Pre-TAVR Anemia Optimization

Although iron deficiency is the most common cause of anemia among TAVR candidates, only a minority of them (~15%) receive iron supplementation before the procedure, and blood transfusions have classically been associated with a greater risk of morbidity and mortality after cardiac interventions [6,10,29]. Few articles have previously addressed treatment strategies for anemia optimization prior to TAVR to minimize the need of transfusions. Urena et al. [30] published in 2017 the only randomized clinical trial in the field to date (EPICURE, Erythropoietin + Iron Therapy for Anemic Patients Undergoing Transcatheter Aortic Valve Replacement). The study, which randomized 100 patients to combined erythropoietin and iron therapy (at days 10 and 1 pre-TAVR) vs. placebo, failed to reduce the need for transfusion or 30-day mortality after TAVR. Despite a non-significant increase in hemoglobin level of 0.2 ± 0.9 g/dL in patients receiving erythropoietin, no differences were observed between study groups in hemoglobin drop post-TAVR or in the rate of procedure-related bleeding complications. The lack of benefit observed in the study might be explained by the limited sample size, high comorbidity burden, or short time between anemia pre-treatment and the procedure. In an observational study, Shuvy et al. [31] pretreated, with either iron or erythropoiesis-stimulating agents, 60 patients with iron deficiency or chronic kidney disease, respectively, 30 days before TAVR with no benefit on 30-day mortality. The unclear benefit of correction of anemia prior to TAVR in these studies outlines the need for alternative, more refined endpoints that enable a more accurate assessment of the potential beneficial impact of correcting anemia before transcatheter procedures.

**Figure 3:**
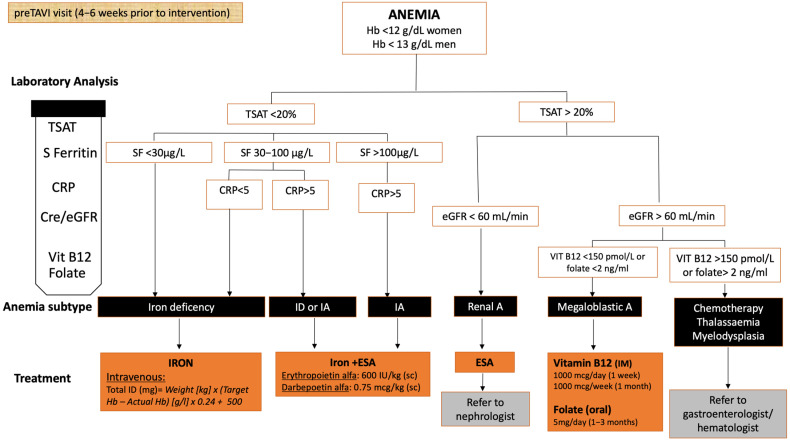
Treatment algorithm for the management of anemia prior to TAVR. Treatment algorithm adapted with permission from Muñoz et al. [33], 2016 © John Wiley and Sons. A: Anemia; CRP: C—Reactive Protein; eGFR: estimated glomerular filtration rate; ESA: erythropoiesis-stimulating agents; Hb: hemoglobin; IA: Inflammatory anemia; ID: Iron deficiency; IM: intramuscular; SC: subcutaneous; SF: Serum ferritin; TSAT: transferrin saturation.

The rationale for preoperative anemia treatment is the optimization of patient hemoglobin levels and iron stores with the ultimate goal of improving patient safety and outcomes. In recent years, patient blood management programs have emerged as an innovative, multidisciplinary approach in cardiac surgery in order to rationalize transfusion rates by optimizing the patients’ red blood cells throughout the perioperative period [32]. Likewise, implementation of specific measures to correct anemia before TAVR may contribute to reducing the need for blood transfusions and improve clinical outcomes. Unfortunately, no randomized trial has shown to date a net clinical benefit of treating pre-operative anemia and iron deficiency before TAVR, and management consensus in this context is lacking. Nonetheless, preprocedural patient optimization whenever possible, especially in elective patients as part of TAVR work-up routines, could help reduce unexpected TAVR complications. We hereby propose a treatment algorithm for the management of pre-operative anemia before TAVR depending on the underlying cause (Figure 3) [33]. The optimal timing for treating preoperative anemia before TAVR remains a matter of debate, but should be initiated as soon as possible and at least 4–6 weeks before the procedure to enable sufficient time to replenish iron stores. Of note, iron deficiency may be preferably corrected with intravenous iron (rather than oral), since oral iron requires at least 3 months to restore iron reserves, and is associated with higher side effects and lower absorption in inflammatory conditions or mixed anemia due to increased hepcidin levels, which inhibit iron absorption from the small bowel and promote iron sequestration [34]. In addition, several studies have shown the advantages of the subcutaneous route of erythropoietin administration (over the intravenous route), in terms of requiring lower dose and frequency of administration without differences in erythropoiesis stimulation, better tolerance, and substantial cost savings [35]. Importantly, caution should be taken when establishing objectives in anemia to avoid overtreatment resulting in iron overload, which may lead to an increased risk of infection or thrombotic events. In the proposed algorithm, the preoperative hemoglobin target was based on the World Health Organization criteria. Whether the threshold for preoperative anemia treatment should be lower in TAVR than in surgical candidates (given the less invasive nature and lower blood loss of transcatheter procedures), as well as the optimal time frame to initiate the preoperative therapy, should be elucidated in future prospective studies. The potential effects of an algorithm-guided management for preoperative anemia in patients undergoing TAVR (using this or alternative algorithms) on perioperative hemoglobin levels and clinical outcomes warrant further investigation. In the meantime, several randomized clinical trials are underway to evaluate whether the correction of iron-deficiency anemia with different intravenous iron supplements (iron isomaltoside NCT04346004, ferric gluconate NCT04797832, ferric carboxymaltose NCT04786769) prior to TAVR procedure may improve clinical outcomes.

## 5. Limitations

This study has the limitations inherent to a systematic review collecting only information described in non-randomized observational studies and, therefore, publication bias cannot be excluded. Six studies were retrospective, and thus it is not possible to control data collection accuracy; missing or confounding data might be included. Although the cause of anemia may be multi-factorial (e.g. iron deficiency, nutritional deficiency, hematological disorders, or chronic disease), the etiology and iron status were only specified in a minority of the studies. Patients with anemia may have had other coexisting non-cardiac comorbidities negatively impacting clinical outcomes or predisposing them to increased mortality, which could not be analyzed given the nature of the study design. Furthermore, the vast majority of studies used a fixed hemoglobin threshold at a specific time point, and very few studies have examined in detail dynamic changes in hemoglobin levels over time or hemoglobin trajectory patterns and their association with outcomes. However, the current study, which included data on >12,000 patients undergoing TAVR, represents the largest and most up to date systematic review on anemic patients scheduled for TAVR.

## 6. Conclusions

Baseline anemia is present in more than one-half of the patients undergoing TAVR and carries an increased risk of transfusion, acute kidney injury, and mortality following TAVR. Although a reversible cause of anemia may be identified in a large proportion of patients scheduled for TAVR, the majority of them remain undertreated. These results provide support for implementation of preventive, targeted approaches for the management of preoperative anemia in patients undergoing TAVR. Future prospective randomized trials assessing the potential benefit of specific measures for anemia optimization prior to TAVR are warranted.

## Figures and Tables

**Table 1:** Clinical outcomes of patients undergoing transcatheter aortic valve replacement according to the presence of baseline anemia (anemia vs. control).

Study (Sample Size)	Follow-Up	Prevalence Anemia (%) ^1^	Age, Yrs	Sex, Male (%)	Hb Level (g/dL)	30-Day			Mid-Term	
						Transfusion	Acute Kidney Injury ^2^	All-Cause Mortality	All Cause-Mortality	All-Cause Rehospitalization
Nuis 2013 [10] (*n* = 1696)	1 year	969 (57%)	81 ± 7 vs. 80 ± 7 (*p* < 0.001)	56% vs. 47% (*p* < 0.001)	11.0 ± 1.1 vs. 13.6 ± 1.0 (*p* < 0.001)	54% vs. 26% (*p* < 0.001)	18% vs. 16% (*p* = ns)	aOR: 1.72 (0.96–3.12)	31% vs. 21% (*p* < 0.001) aHR: 1.42 (1.12–1.81)	-
De Backer 2014 [19] (*n* = 253)	2 years	124 (49%)	80 ± 7 vs. 79 ± 7 (*p* = 0.895)	66% vs. 56% (*p* = 0.121)	11.8 vs. 13.7	28% vs. 16% (*p* = 0.033)	6% vs. 5% (*p* = 1.000)	-	16% vs. 10% (*p* = ns)	OR: 1.75 (1.03–2.98)
Arai 2015 [9] (*n* = 3472)	1 year	1335 (38.5%)	S ^3^ 82.1 ± 8.8 M 82.7 ± 10.1 C 82.7 ± 7.7 (*p* = 0.30)	S ^3^ 58% M 51% C 49% (*p* <0.01)	S ^3^ 9.6 ± 0.7 M 11.1 ± 0.4 C 13.1 ± 1.1 (*p* <0.01)	S ^3^ 26% M 21% C 15% (*p* < 0.01)	S ^3^ 10% M 8% C 5% (*p* < 0.01)	S ^3^ 10% M 7% C 6% (*p* = 0.02)	aHR: 1.44 (1.28–1.63)	-
De Larochellière 2015 [6] (*n* = 438)	6 months	282 (64.4%)	80 ± 8 vs. 78 ± 9 (*p* = 0.020)	51.8% vs. 43.6% (*p* = 0.101)	10.8 ± 1.1 vs. 13.4 ± 1.0 (*p* < 0.001)	39.7% vs. 20.0% (*p* < 0.001)	14.2 vs. 21% (*p* = ns)	6% vs. 7.7% (*p* = ns)	-	-
Hellhammer 2016 [20] (*n* = 376)	1 year	239 (63.6%)	82 ± 6 vs. 81 ± 6 (*p* = 0.101)	46.9% vs. 40.1% (*p* = 0.207)	11.0 ± 1.1 vs. 13.6 ± 1.1 (*p* < 0.001)	-	25.1% vs. 10.9% (*p* = 0.001)	9.2% vs. 3.6% (*p*= 0.045)	-	-
Seiffert 2017 [21] (*n* = 1201)	3 years	707 (59%)	82 (76–86) vs. 82 (77–85) (*p* = 0.854)	53.3% vs. 41.3% (*p* = 0.001)	-	48.8% vs. 18.1% (*p* < 0.001) OR: 6.74 (4.8–9.6)	9.6 vs. 4.9% (*p* = 0.003)	9.5% vs. 8.8% (*p* = 0.626) HR: 1.50 (0.88–2.55)	64.9 vs. 49.6% (*p* = 0.002) aHR: 1.43 (1.13–1.82)	-
Rheude 2017 [18] (*n* = 549)	1 year	249 (45%)	82 ± 6 vs. 80 ± 6 (*p* < 0.001)	55% vs. 54% (*p* = 0.947)	11.0 ± 1.1 vs. 13.6 ± 1.1 (*p* < 0.001)	25% vs. 4% (*p* < 0.001)	6% vs. 1% (*p* = 0.005)	-	14% vs. 3% (*p* < 0.001)	16% vs. 6% (*p* < 0.001) ^6^
Nicolas 2020 [15] (*n* = 877)	1 year	465 (53%)	S ^4^ 83.1 ± 5.9 M 82.7 ± 6.3 C 82.1 ± 6.4 (*p* = 0.26)	0%	S ^4^ 9.0 ± 1.1 M 11.1 ± 0.6 C 13.2 ± 1.0 (*p* < 0.001)	S ^4^ 17.9% M 5.2% C 3.5% (*p* < 0.001)	S ^4^ 1.0% M 1.9% C 1.2% (*p* = ns)	S ^4^ 3.9% M 3.9% C 3.2% (*p* = ns)	S ^4^ 20.7% M 12.8% C 9.5%. (*p* = 0.002)	-
Razuk 2021 [17] (*n* = 798)	30 days	427 (54%)	82.7± 6.7 vs. 81.8 ± 6.3 (*p* = 0.070)	54.1% vs. 48.0% (*p* = 0.085)	11.2 ± 1.7 vs. 13.3 ± 1.8 (*p* = 0.001)	-	20.4% vs. 12.1% (*p*= 0.001) OR: 1.74 (1.16–2.59)	-	-	-
Bastug 2021 [22] (*n* = 340)	2.5 years	170 (50%)	79 (73–84) vs. 78 (72–83) (*p* = 0.33)	42.4% vs. 46.5% (*p* = 0.44)	10.9 (10.2–11.6) vs. 13.4 (12.7–14.1) (*p* < 0.001)	-	-	-	53.5% vs. 27.1% (*p* < 0.001) HR:2.58 (1.77–3.75)	-
Onoda 2021 [16] (*n* = 2588)	2 years	1872 (72.3%) Onoda cut-off ^5^: 909 (35.1%)	85.3 ± 5.2 vs. 83.9 ± 5.2 (*p* < 0.001)	29.7% vs. 32.2% (*p* = 0.024)	9.5 ± 0.8 vs. 12.2 ± 1.2 (*p* < 0.001)	42.8% vs. 21.6% (*p* < 0.001)	15.2% vs. 9.0% (*p* < 0.001)	4.0% vs. 2.0% (*p* = 0. 004)	17.9% vs. 13.2% (*p* < 0.001) HR: 1.77 (1.39–2.25)	-

Data are expressed as *n* (percentage), mean ± SD, or median (interquartile range). Abbreviations: aHR: adjusted hazard ratio; aOR: adjusted odds ratio; C: control; Hb: Hemoglobin; HR: hazard ratio; M: moderate anemia; ns: non-significant; OR: odds ratio; S: severe anemia; U: units of red blood cells. ^1^ World Health Organization anemia classification (Hb < 12.0 g/dL in women and <13.0 g/dL in men). ^2^ Acute kidney injury within 7 days post-procedure according to VARC-2 criteria. ^3^ Anemia classification (tertiles): S (severe): Hb < 10.23 g/dL in women and <10.80 g/dL in men; M (moderate): Hb 10.23–11.29 g/dL in women and 10.80–11.99 g/dL in men; C (control with no or mild anemia): Hb ≥ 11.30 g/dL in women and ≥12.0 g/dL in men. ^4^ Anemia classification (tertiles): S (severe): Hb < 10 g/dL; M (moderate/mild): 10–12 g/dL; C (control): Hb ≥ 12 g/dL. ^5^ Onoda cut-off anemia value: Hb < 10.4 g/dL in women and <10.9 g/dL in men. ^6^ Hospitalization due to heart failure.

## Data Availability

Not applicable.

## Supplementary Materials

The following supporting information can be downloaded at: https://www.mdpi.com/article/10.3390/jcm12186025/s1, Table S1: PRISMA checklist for systematic reviews. Table S2: Acute kidney injury classification according to the Valve Academic Research Consortium 1 and 2. Table S3: Summary of methodological quality analysis using QUIPS tool for each prognostic study included in the review. Figure S1: Sensitivity Analysis 30-day need of transfusion.
